# Nutrition of Children and Women in Bangladesh: Trends and Directions for the Future

**DOI:** 10.3329/jhpn.v30i1.11268

**Published:** 2012-03

**Authors:** Tahmeed Ahmed, Mustafa Mahfuz, Santhia Ireen, A.M. Shamsir Ahmed, Sabuktagin Rahman, M. Munirul Islam, Nurul Alam, M. Iqbal Hossain, S.M. Mustafizur Rahman, M. Mohsin Ali, Fatima Perveen Choudhury, Alejandro Cravioto

**Affiliations:** ^1^Centre for Nutrition and Food Security, icddr,b, GPO Box 128, Dhaka 1000, Bangladesh; ^2^James P. Grant School of Public Health, BRAC University, Mohakhali, Dhaka 1212, Bangladesh; ^3^National Nutrition Services, Dhaka, Bangladesh; ^4^UNICEF Bangladesh, Dhaka, Bangladesh; ^5^Institute of Public Health Nutrition, Mohakhali, Dhaka 1212, Bangladesh

**Keywords:** Anaemia, Child nutrition, Maternal nutrition, Nutrition disorders, Review literature, Bangladesh

## Abstract

Although child and maternal malnutrition has been reduced in Bangladesh, the prevalence of underweight (weight-for-age z-score <-2) among children aged less than five years is still high (41%). Nearly one-third of women are undernourished with body mass index of <18.5 kg/m^2^. The prevalence of anaemia among young infants, adolescent girls, and pregnant women is still at unacceptable levels. Despite the successes in specific programmes, such as the Expanded Programme on Immunization and vitamin A supplementation, programmes for nutrition interventions are yet to be implemented at scale for reaching the entire population. Given the low annual rate of reduction in child undernutrition of 1.27 percentage points per year, it is unlikely that Bangladesh would be able to achieve the United Nations’ Millennium Development Goal to address undernutrition. This warrants that the policy-makers and programme managers think urgently about the ways to accelerate the progress. The Government, development partners, non-government organizations, and the academia have to work in concert to improve the coverage of basic and effective nutrition interventions, including exclusive breastfeeding, appropriate complementary feeding, supplementation of micronutrients to children, adolescent girls, pregnant and lactating women, management of severe acute malnutrition and deworming, and hygiene interventions, coupled with those that address more structural causes and indirectly improve nutrition. The entire health system needs to be revitalized to overcome the constraints that exist at the levels of policy, governance, and service-delivery, and also for the creation of demand for the services at the household level. In addition, management of nutrition in the aftermath of natural disasters and stabilization of prices of foods should also be prioritized.

## INTRODUCTION

The term malnutrition refers to both undernutrition and overnutrition. Undernutrition encompasses protein-energy malnutrition and deficiency of micronutrients, including essential vitamins and minerals. Undernutrition is the underlying cause of 3·5 million deaths and 35% of the burden of diseases among children aged less than five years (under-five children) worldwide (1). Of the total global disability-adjusted life-years (DALYs), 11% are due to childhood malnutrition alone. About 80% of undernourished children of the world live in just 20 countries in Africa, Middle East, Asia, and the Western Pacific; Bangladesh is one of these countries (2). In Bangladesh, undernutrition continues to be a serious public-health problem. Although overnutrition is still not a large problem, the prevalence of overweight among under-five children and women is increasing. The percentage of children with weight-for-height or body mass index (BMI) z-scores ≥3 was 0.1 in 1995 (3), which has increased to 0.5% according to a recent national survey (4). Similarly, in 1996-1997, 2.7% of women were overweight/obese (BMI *≥* 25 kg/m^2^), which has increased to 10.1% in 2007 (4,5). However, the issue of overnutrition is beyond the scope of this paper. The paper summarizes the trends in undernutrition situation in Bangladesh, explores the potential reasons for slow improvements of the nutrition situation, and attempts at charting out directions for the future.

## MATERIALS AND METHODS

The methodology involved desk reviews of published results of various national-level nutrition surveys conducted by the Government and non-government organizations (NGOs). These surveys included, among others, the Bangladesh Demographic and Health Surveys since 1990 up to 2007 and the nutrition surveys conducted by Helen Keller International and United Nations Children's Fund (UNICEF).

## RESULTS

### Protein-energy malnutrition

**Fig. 1.**
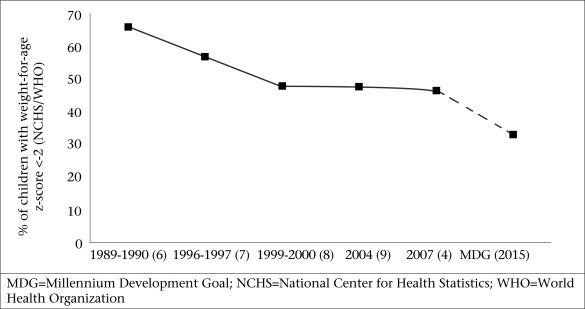
Trends in prevalence of underweight among under-five children in Bangladesh

Undernutrition has been and continues to be a serious public-health problem in Bangladesh (4). The prevalence of underweight (weight-for-age z-score <-2) among under-five children in 1989-1990 was more than 65% (Fig. 1). Although it came down to 47% in 2000, little change has been observed since then. The annual rate of reduction in underweight required to achieve the nutrition target of Millennium Development Goal (MDG) 1 is 1.36 percentage points while the current rate is 1.27 percentage points per year. This low rate of reduction in the prevalence of underweight makes it unlikely for the country to achieve the nutrition target of MDG 1. However, even if we achieve the target, the prevalence of underweight will still be unacceptably high (>33%). Furthermore, 43% of under-five children are still stunted (height-for-age z-score <-2), and wasting is present in 17% of children, with 3.4% being severely wasted (4,10). This accounts for more than 0.5 million children with severe acute malnutrition (SAM) in the country. These children who are suffering from various forms and degrees of undernutrition are at a high risk of death or severe impairment of growth and development (11).

Unlike children, the nutritional status of women in Bangladesh shows a better trend. In 1997, 52% of women had chronic energy deficiency (CED), defined as BMI <18.5 kg/m^2^. Since then, a sustained reduction has been observed in the prevalence of CED; the prevalence was 30% in 2007 (4).

### Low birthweight

Low birthweight (LBW—birthweight of <2,500 g) is an important indicator of the overall health of the mother and the newborn. This practical cut-off of LBW, defined by the World Health Organization (WHO), is based on epidemiological observations that the odds of a baby dying who weighs <2,500 g at birth is 20 times greater than for a heavier baby (12). Intrauterine growth retardation (IUGR) and preterm birth (PTB) are the two main causes of LBW. PTB is defined as gestational age of <37 weeks at delivery. In low-income countries, the majority of LBW infants are born small but not premature (13). A study in rural Bangladesh, using the Parkin method, to determine gestational age, reported that IUGR was the major contributor to LBW (96.4%) while only 3.6% of babies were born preterm (14). The global prevalence of LBW is 15.5%, with developing countries accounting for more than 95% (15). The rates of LBW among Bangladeshi infants, though reduced from 40%, are still among the highest in the world, ranging from 20% to 22% (16-18). After controlling for the independent effects of other covariates, maternal BMI and height were shown to be the powerful predictors of LBW in Bangladesh (16).

### Micronutrient deficiencies

Micronutrient-related malnutrition is often termed ‘hidden hunger’ as the consequences are not always visible. There are four micronutrients that are particularly relevant to public health: vitamin A, iron, iodine, and zinc. The following sections briefly describe the situation of micronutrient deficiencies in Bangladesh.

#### Vitamin A deficiency

**Fig. 2.**
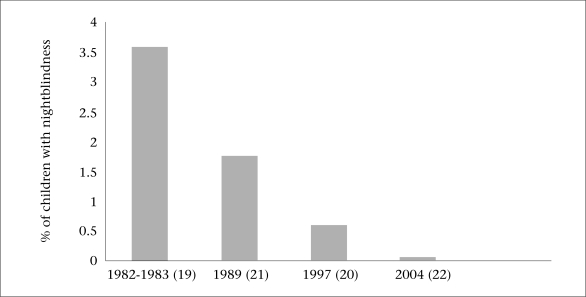
Trends in prevalence of nightblindness among preschool children in Bangladesh

In Bangladesh, vitamin A deficiency (VAD) has been identified as a major public-health problem in the last two decades (19). There has been a dramatic reduction in the prevalence of nightblindness among preschool children from the 1980s to 2004, which is attributed to the successful programme of vitamin A supplementation launched in 1973 (Fig. 2) (19-22). Keratomalacia, the most severe form of VAD, is now seen occasionally among children hospitalized for SAM. However, in a recent study in rural Bangladesh, sub-clinical VAD (serum retinol <0.7 µg/dL) was found in 18.5% of 200 pregnant women (23). The vitamin A intake by nearly half of pregnant women was less than the recommended dietary allowance.

In Bangladesh, children aged 9-11 months receive vitamin A capsules (100,000 IU) at the time of measles vaccination, and children aged 12-59 months receive a capsule of 200,000 IU every six months. The Bangladesh Demographic and Health Surveys (BDHS) collect information on children receiving one capsule during the last six months, and the latest BDHS report (2007) showed an impressive coverage of immunization at 88% (4). The coverage of postpartum vitamin A supplementation to mothers, however, is very low (20%) (4).

#### Iron deficiency

Anaemia is the most commonly-used indicator to define iron deficiency in population-based studies or in clinical settings. It is generally assumed that 50% of anaemia cases are due to iron deficiency; however, acute and chronic infections, including malaria, cancer, tuberculosis, and HIV, can also lower the blood haemoglobin levels (24). The presence of other micronutrient deficiencies, including vitamins A and B_12_, folate, riboflavin, and copper, increases the risk of anaemia. The main risk factors for iron-deficiency anaemia include a low intake of iron, poor absorption of iron from diets high in phytate or phenolic compounds, and periods when iron requirements are especially high, such as periods of rapid growth during childhood and pregnancy. Among the other causes of anaemia, heavy blood loss as a result of menstruation, or para-site infestations, such as hookworm or *Ascaris*, are noteworthy. The population groups with the highest risk for iron deficiency are preterm and LBW infants, children during periods of rapid growth, premenopausal women, pregnant women, and individuals with parasite infestations in the gastrointestinal tract (24).

**Fig. 3.**
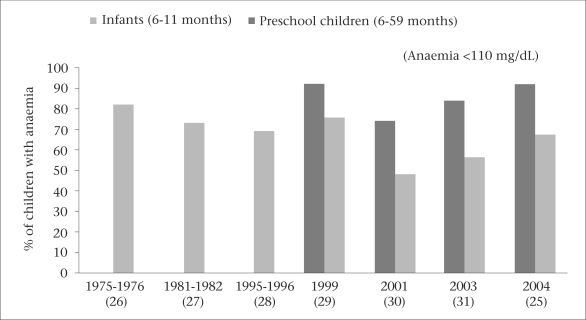
Trends in prevalence of anaemia among infants and preschool children in Bangladesh

Surveys in 2003-2004 showed that 92% of infants aged 6-11 months in Bangladesh suffer from anaemia (Fig. 3) (25). Its prevalence remains high among preschool children (68%) and adolescent girls (40%). Four in 10 pregnant women still suffer from anaemia, particularly in rural areas. The prevalence of anaemia increased during 2001 to 2004 (25). This high burden of anaemia impacts on the economy of the country, and an estimated 7.9% of gross domestic product (GDP) in Bangladesh is lost due to anaemia alone (25).

The persistent high prevalence of anaemia among both children and women indicates that there are gaps and challenges in the existing strategies. The programme for the supplementation of iron-folic acid (IFA) to pregnant women is active but the coverage is only 55% (4). And for infants and preschool children, despite the frightening prevalence of anaemia and its potential consequences on cognitive functions, no interventions have been undertaken to control the situation at scale. However, some small-scale projects by NGOs are currently providing micronutrients for home fortification of foods.

#### Iodine deficiency

Iodine deficiency is one of the most important causes of preventable brain damage in children. In 1993, the prevalence of goitre in Bangladesh was 47.1%, cretinism 0.5%, and sub-clinical iodine deficiency (low urinary concentration of iodine <100 µg/L) 69% (32). The latest Iodine Deficiency Disorder (IDD) Survey showed that the prevalence of goitre among 6-12 years old children was 6.2%, and it was 11.7% among women aged 15-44 years (33). However, more than one-third of children and women were suffering from sub-clinical iodine deficiency. The IDD survey reported that, in 2004-2005, more than 80% of households used salt which was poorly iodized (<15 parts per million–ppm but ≥5 ppm), and nearly 50% of households used salt which was adequately iodized (≥15 ppm) (33). Another recent national survey revealed that more than 40% of households used iodized table salt that had low iodine (<15 ppm) (16). This calls for improving the quality of salt iodization and its coverage.

#### Zinc deficiency

At the population level, the risk of zinc deficiency can be assessed based on two indirect indicators: (a) the prevalence of stunting and (b) the adequacy of absorbable zinc in food supply at the country level (1). A stunting rate of more than 20% in under-five children is indicative of high risk for zinc deficiency at the country level (1). With a 43% prevalence of stunting among under-five children, zinc deficiency is a major nutritional disorder in Bangladesh. The beneficial effects of zinc treatment during diarrhoea are well-known (34). Meta-analyses of rando-mized controlled trials of zinc for the treatment of diarrhoea and respiratory illness estimated that the overall impact of zinc treatment in under-five children results in a 15% reduction in the duration of illness, a 16% decreased likelihood of progressing to a severe episode, and a 25% reduction in persistent childhood diarrhoea. Additionally, children who had received zinc treatment experienced about 15% fewer repeat episodes of diarrhoea during the next three months (35-37). There are 18.9 million under-five children in Bangladesh, and it is estimated that zinc treatment during diarrhoea could save the lives of 30,000-75,000 children per year in Bangladesh (38). Zinc as an adjunct therapy for the treatment of diarrhoea is currently being promoted by both public and private sectors in Bangladesh. Compared to other countries, the use of oral rehydration therapy (ORT) plus zinc during diarrhoea is the highest in Bangladesh, although only one in five children currently receives zinc with ORT during diarrhoea.

### Breastfeeding and complementary feeding practices

Proper feeding practices during infancy and childhood are essential for attaining and maintaining proper nutrition and health, and for development of infants and children (39). The WHO recommends exclusive breastfeeding (EBF) for the first six months of life and continuation of breastfeeding for two years. EBF in the first six months and continued breastfeeding for the next 6-11 months have been estimated to prevent 13% of all under-five deaths in the developing world (38). Compared to EBF, not breastfeeding is associated with a 14-time higher risk of death due to any cause in 0-5 month(s) old children (1).

**Fig. 4.**
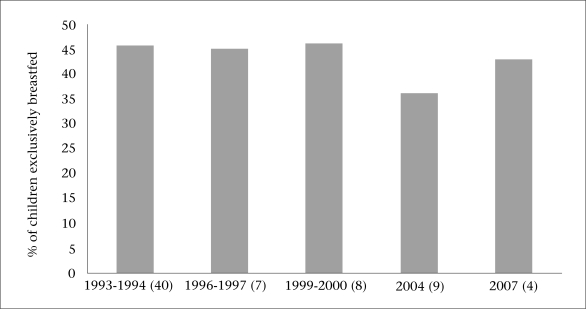
Trends in exclusive breastfeeding among children aged less than six months in Bangladesh

Breastfeeding is common in Bangladesh; 99% of infants aged less than 12 months are breastfed. However, the prevalence of EBF is 43% in infants aged less than six months, and this has not changed over the last decade (Fig. 4), with serious implications for the nutritional well-being and mortality of children (4). Forty-three percent of neonates are breastfed within one hour of birth. The median duration of breastfeeding is 32.8 months while that of EBF is only 1.8 months.

Inappropriate feeding practices, particularly after the age of six months when breastmilk alone is no longer sufficient to meet the increasing nutrient requirements for growth, result in high rates of childhood undernutrition in developing countries. In Bangladesh, complementary feeding starts too early or too late, and foods that are offered are often inappropriate, leading to high rates of childhood undernutrition. Even among infants aged less than two months, 17% drink milk other than breastmilk (fresh or powdered cow's milk, infant formula, etc.); 6% are given other liquids; and another 6% receive solid or semi-solid foods (4). On the other hand, 25% of infants aged 6-9 months are not fed any solid or semi-solid foods in addition to breastmilk. Of the older children who have crossed exclusive breastfeeding age, only 42% are fed according to the recommended infant and young child-feeding practices. Furthermore, results of a study in Bangladesh showed that the amount of energy from complementary foods offered to infants was about 74% of the recommended amount (41). The mean intakes of vitamin A from breastmilk and complementary food together were 44% and 48% of the required nutrient intakes for children aged 6-8 months and 9-12 months respectively. The intakes of vitamin D and zinc were 12-13% of the recommended nutrient intake (RNI) and 40-45% respectively. The intake of iron was very low, accounting for only 8-9% of the RNI. The findings of the study imply that it is difficult to improve the micronutrient intakes of children by simply increasing the amount of complementary food currently consumed in Bangladesh. Other interventions, including home fortification of food or a complementary food for children that has added micronutrients and other nutrients require special consideration.

### Why has reduction in child undernutrition slowed down in Bangladesh?

Bangladesh has seen an impressive two-percentage point decline per year in the prevalence of underweight among under-five children from the 1990s to 2000, which is potentially accounted for by both improved social and health-system indicators. Improvement in wealth and maternal education (up to secondary level) had the strongest effects on reducing child undernutrition in the late 1990s among other contributing factors, such as reduced fertility rate, measles immunization, and rural electrification (42). The female secondary literacy rate went up from 2.1% in 1993-1994 to 25.6% in 1999-2000 (40,8). In 1990-1991, 9,847 villages were connected to the electricity grid while, in 2008-2009, this number increased to 47,641 (43). Measles immunization skyrocketed from a mere 2% in 1985 to 76% in 2000, and the coverage of vitamin A supplementation almost doubled to 84% (8). These factors presumably have exerted a positive impact on nutrition by improving the feeding and care practices, thereby affecting the immediate and underlying causes of undernutrition (44). The production of rice increased from 18.2 million metric tonnes in 1990-1992 to 24.1 million metric tonnes in 1999-2001 (45). This period also witnessed a reduction in the fertility rate and family-size, which may have positively affected the nutritional status of children. The total fertility rate reduced from 4.3 in 1989-1991 to 3.3 in 1997-1999 (4). Notwithstanding the impressive improvements, the reduction in childhood undernutrition has plateaued from 2000 onwards (Fig. 1). However, it is difficult to establish any causal relationship considering that undernutrition is a multifaceted complex phenomenon, involving many immediate, underlying and structural factors. Nevertheless, some assumptions can be drawn on the underlying and immediate causes for this stagnation in rates of childhood undernutrition.

Reduction in poverty leads to improvement in the nutritional status, although increase in income inequality has a negative impact on the nutritional status of under-five children and women of child-bearing age (46,47). Haddad *et al*. have shown that increase in income at the household and national levels can significantly improve the nutritional status, although growth of income alone would be unable to help meet the nutrition target of MDG unless it is coupled with direct nutrition interventions (48). In Bangladesh, when nutritional status was modelled against GDP per capita for the last three decades (up to 2000), reduction of 23.1% and 22.4% in stunting and underweight respectively could be explained by GDP growth alone. However, when income inequality was added to the model, the nutritional status (stunting) improved up to a certain level of income (over US$ 2,200 per capita per year) but after this level, inequality had a negative impact on nutrition (42). The likely explanation for this result could be that, at lower incomes, if resources are equally distributed, everyone is malnourished. However, with inequality, some might escape undernutrition while others are pushed further into food deprivation and consequent undernutrition. Similarly, results of analysis of data from the BDHS 2004 showed that children aged 0-59 months in the poorest 20% of households were more than three times as likely to be stunted as children from the wealthiest 20% of households (odds ratio=3.6; 95% confidence interval 3.0-4.3) (stunting rates in the poorest and wealthiest quintiles were 55.4% and 25% respectively) (46).

Bangladesh has made a good progress in increasing the per-capita income, particularly since the second half of the 1990s when its growth rate accelerated to 3.6% per year (49). There has been a reduction in absolute poverty (<2,122 kcal per capita per day) from 47.5% in 1991-1992 to 31.5% in 2010 (50,51). However, the income share of the lower-tier people has decreased while that of the top increased by 50%, widening the gap between the richest and the poorest. The income Gini coefficient (which measures income inequality and a greater value represents greater inequality) shows an increase from 0.432 in 1995 to 0.451 in 2000 and to 0.458 in 2010 mostly because of increasing inequality between urban and rural areas (50-52). Results of analysis of the food-consumption pattern over the years revealed a decline in average food consumption and intake of calorie and protein in 2000 compared to 1995-1996 (50). Therefore, this income inequality between the rich and the poor, and between urban and rural populations, and the decline in food consumption could, in part, explain the slow improvements in nutritional status after 2000. The consumption pattern has, however, improved in 2005 compared to 2000 (50).

Infant mortality has dramatically decreased from 133 deaths per 1,000 livebirths in 1989-1993 to 65 deaths per 1,000 livebirths in 2002-2006, which could partly be due to successful programmes, such as immunization and vitamin A supplementation (4). Infants who survived are likely to grow up and become malnourished in the absence of proper feeding and caring practices. Furthermore, the prevalence of acute malnutrition (weight-for-height z-score <-2) has also increased from 10% in 1999-2000 to 17% in 2007, which is much higher than the critical level that warrants a public-health intervention (53). Although most of these malnourished children could be managed at the community level, Bangladesh is yet to develop and implement a community-based programme to tackle this high level of acute malnutrition. The persistence of high rates of acute malnutrition is also contributing to the high burden of and slow reduction in childhood undernutrition.

## WAY FORWARD

Adequate nutrition is a prerequisite for attaining good health, quality of life, and national productivity. The current low rate of decline in undernutrition and the marked increase in prices of rice and other essentials over the last couple of years raise concerns about the country's food security and nutrition situation and, in turn, about achieving the nutrition target of MDG 1. Bangladesh is placed in the bottom 25% of the Global Hunger Index ranking, indicating that the country is expected to face a huge risk in the context of food price-hike (10). The Household Food Security and Nutrition Survey, assessing the impact of price-hike shock during 2007-2008 on the food security and nutrition situation revealed that, to cope with the high prices of food, alongside reducing portion-sizes and borrowing money, 22% of families also reduced health expenditure, which is likely to have impacted on health (10). Nevertheless, the retail price of rice during the early part of 2011 was 3% higher than its peak in the food price shock year of 2008, which indicates a greater risk than that of 2008 faced by children and women (54).

The major challenges to improving the nutrition situation in Bangladesh warrant critical planning and significant investments in appropriate interventions, integrating both direct and indirect routes for improvement. Direct actions will first require a sound policy framework and a national plan of action, followed by scaling up of effective nutrition interventions.

**Policy framework for nutrition:** Nutrition interventions are currently being delivered in a sporadic manner. The largest single provider of comprehensive nutrition interventions targeting adolescent girls, women, and children was the National Nutrition Programme (NNP) administered by the Government and implemented by local NGOs. Although the programme was based on a robust design tailored to local needs, it covered only about 30% of the total population, and its efficacy has been questioned (42,55,56). The targeting of the programme beneficiaries was not appropriate while the food supplement provided to children and women was of low nutritional quality. Accountability in the programme was sub-optimal and, moreover, the monitoring and evaluation mechanisms were weak. The programme did not cater to the needs of children with SAM, and it did not cover any of the cities which have substantial slum populations. In the light of these weaknesses, the Government, in the new Health, Population and Nutrition Sector Development Programme (HPNSDP, 2011-2016), has closed the NNP and has decided to mainstream the nutrition services into the health system.

In view of mainstreaming the nutrition services into the health system, it is important that the existing knowledge and learning from the NNP is taken up and revisions are made in the approach where necessary. The revision should focus on improved and effective behaviour change communication, improved micronutrient status through food intake and through micronutrient supplementation, better complementary feeding, etc. Food supplementation should not be routine but may be considered only safety-net for affected populations living in nutrition and food-insecurity hotspots in the country. Furthermore, the quality and quantity of the supplement should be revisited and revised to make it nutritionally appropriate by adding micronutrients and any foods from animal source, such as milk, which is vital for the growth of malnourished children. At the community level, the basic nutrition interventions will now be provided through the community clinics which the Government plans to establish country-wide—one clinic for 6,000 people. For this model to be effective, a doctor working at the sub-district health complex may be assigned as the nutrition manager; he/she will oversee the programme at the sub-district level, supported by field supervisors and community health workers. However, it is important that there should be one person at the community clinic dedicated to nutrition interventions. Otherwise, there is a risk that nutrition might lose priority among many competing health issues. This will clearly require mobilization of additional, trained personnel. For tackling the mammoth problem of undernutrition, synchronized inter-ministerial activities are essential. Coordination between various ministries that play important roles in health, nutrition and food security and also with the NGOs, the private sector, and the international initiatives on nutrition issues (Scaling up Nutrition, REACH, and Feed the Future) can only be warranted by a high-powered body in the Prime Minister's Office.

**Scaling up of effective nutrition interventions:** Since a single intervention would make a little difference, we need several interventions implemented at scale through the public sector and NGOs. A list of evidence-based interventions that should be implemented in the high-burden countries was published recently (34). Interventions that would tackle the direct and immediate factors of undernutrition should target not only the ‘window of opportunity’ or the first 1,000 days (the period between conception and up to two years of age) but also the period before that—the adolescent period as part of the life-cycle approach. The interventions targeting undernutrition should be scaled up to cover at least 70% of the total population to show tangible outcomes. The priority interventions for scaling up in Bangladesh should include the following:

### During pregnancy and lactation

- Supplementation of iron-folic acid tablets to mothers to combat anaemia during pregnancy and lactation
- Effective counselling for increased rest and food intake during pregnancy and counselling on appropriate infant-feeding practices during the second half of pregnancy
- Regular consumption of iodized salt
- Supplementation of a dose of vitamin A (200,000 units) to mothers within six weeks of delivery

### 0-5 months

- Encouragement of EBF during early infancy through individualized counselling and trouble-shooting for the challenges faced during breastfeeding
- Creating an awareness of the importance of breastfeeding through multiple channels, such as classroom discussions for adolescent girls, counselling during pregnancy, feeding support, and trouble-shooting during the first few hours and days after child birth, and media coverage

### 6-23 months

- Promotion of continued breastfeeding
- Counselling of mothers on complementary feeding using energy-dense local foods made of cereals, vegetables, oil, lentils, and, whenever possible, animal protein (fish/egg/meat)
- Six-monthly supplementation of vitamin A
- Zinc treatment and ORT during diarrhoea
- Hygiene interventions, including handwashing
- Multiple micronutrient powder for home fortification of food
- Deworming according to the guidelines of WHO
- Treatment for SAM at the facility and community levels with ready-to-use-therapeutic foods made from local food ingredients

### Adolescent girls and newly-married women

- Supplementation of iron-folic acid tablets
- Nutrition and health education
- Deworming

Since around 40% of the population cannot afford to have the desired number of calories per day, food security is a key to improving nutrition. A multi-sectoral approach for interventions that indirectly impact on food security and nutrition should include promotion of pro-poor livelihood opportunities, such as direct cash or productive asset transfers, women empowerment, education of girls, safe water, improvement in agricultural and livestock, poultry production, etc.

### Conclusions

The key challenges for promoting programmes to prevent undernutrition at the national level in Bangladesh include: placing nutrition high up on the list of priorities, implementing cost-effective and sustainable interventions at scale following appropriate strategies, improving access to the servi-ces for those in real need, and evidence-based decision-making and building up operational capacity. In addition to health and nutrition interventions, economic and social policies addressing poverty, trade, and agriculture that have been associated with rapid improvements in nutritional status should be implemented. Nevertheless, for any nutrition and health intervention to be cost-effective, strong governance is essential. Failing to respond to the population's need and provide the right services to the right people and weak accountability at all levels of designing through implementation and evaluation would result in unsuccessful efforts.

## ACKNOWLEDGEMENTS

This research was funded by icddr,b and Mainstreaming Nutrition Initiative funded by the World Bank. icddr,b also gratefully acknowledges the following donors which provide unrestricted support: Government of the People's Republic of Bangladesh, Canadian International Development Agency (CIDA), Embassy of the Kingdom of the Netherlands (EKN), Swedish International Development Cooperation Agency (Sida), and the Department for International Development (DFID), UK.
